# Changes and events over life course: a comparative study between groups of older adults

**DOI:** 10.1590/0104-1169.0144.2518

**Published:** 2015

**Authors:** Luípa Michele Silva, Antônia Oliveira Silva, Luiz Fernando Rangel Tura, Maria Adelaide Silva Paredes Moreira, Jordana Almeida Nogueira, Stefano Cavalli

**Affiliations:** 1 Doctoral student, Universidade Federal da Paraíba, João Pessoa, PB, Brazil. Scholarship holder from Coordenação de Aperfeiçoamento de Pessoal de Nível Superior (CAPES), Brazil; 2 PhD, Associate Professor, Universidade Federal da Paraíba, João Pessoa, PB, Brazil; 3 PhD, Associate Professor, Universidade Federal do Rio de Janeiro, Rio de Janeiro, RJ, Brazil; 4 PhD, Professor, Universidade Federal da Paraíba, João Pessoa, PB, Brazil; 5 PhD, Professor, Center of Competence on Aging, DEASS, University of Applied Sciences and Arts of Southern Switzerland (SUPSI), Switzerland

**Keywords:** Life Change Events, Aged, Aged, 80 and Over

## Abstract

**OBJECTIVE:**

to identify the changes which had occurred over the last year in the life of older adults, as well as the values attributed to these changes.

**METHOD:**

this is a multicentric, cross-sectional study, of the inquiry type, undertaken in three cities of the Brazilian Northeast, investigating two distinct groups of older adults.

**RESULTS:**

among the 236 older adults interviewed, it was observed that 30.0% reported health as the main change in their life course in the last year, this category being the most significant response among the older adults aged between 80 and 84 years old (37.7%). Changes in the family were mentioned by 11.5% of the older adults; death (9.6%) and alterations in routine activities (9.6%). In relation to the value attributed to these changes, it was ascertained that for 64.7% of the older adults aged between 65 and 69 years old, these changes were positive. In the older group, 49.4% of the older adults believe that their changes were related to losses.

**CONCLUSION:**

the knowledge of the changes mentioned, the value attributed to these changes, and the self-evaluation of health provide information which assists in formulating actions which are more specific to the real needs of these age groups. They also provide the health professionals with a better understanding of how some experiences are experienced in the life trajectories of these older adults.

## Introduction

Understanding the continuity of the changes which have occurred during the life course is one of the principal theoretical-methodological challenges for contemporary gerontology, although there is no conceptual consensus regarding their meaning. In the last 50 years, five different uses of the term 'life course" have been identified: "time or age", "life stages", "events, transitions and trajectories", "life-span human development", "influences in the beginning of life (and their accumulation) on the results of later adults"^(^ 1 ^)^.

Although its multiplicity of meanings involves a rich variety of formulations and conceptions, this study incorporates the concept of "life course", understanding it as being "events, transitions and trajectories of life".

The notion of change in one's own life or around one is based on the subjective perception which individuals have of these occurrences, and how they value them. The way in which each individual perceives his own trajectory is unique, differentiated and influenced by the regional culture, beliefs and ways of life observed in society. Positive or negative interpretations of certain events experienced result from unique experiences and extend to physical, mental and social health^(^ 2 ^)^.

In daily life, predictable occurrences, such as marriage, or unpredictable ones, such as the death of a family member or unemployment, can have varying effects on subjective well-being. The strength of these effects and the consequences on the individual differ according to the value that each person attributes to the same^(^ 3 ^)^. Hence, the individual trajectories are of fundamental importance in how the individuals see the transitions in their life journey, and how they interfere in the process of life and illness in different contexts^(^ 3 ^-^ 4 ^)^.

In this perspective, the older adults were chosen so as to represent the population to be studied, due to their experiences and their life histories, and because they were in a process of change involving, for example, the adjustment to retirement in the maintenance of activities of daily living, such as social and civic activities^(^ 5 ^)^.

This study's aim is not restricted to a simple exhaustive surveying of facts regarding all the changes and occurrences experienced by the older adults or those around them, but focusses on aspects particularly significant to the same. It is anchored in an international study termed Changes and Events across the Life Course (*Changements et événements au cours de la vie*) (CEVI), devised in Geneva in 2003 by Prof. Christian Lalive d'Epinay and Prof. Stefano Cavalli; which was extended to which was extended to Argentina (2004), Mexico (2005), Canada (2007), Chile (2008), Belgium, France and Italy (2009), Brazil (2010), and Uruguay (2012).

CEVI has, as its scope of investigation, the representations of the life trajectories of adult individuals from various countries, understood as the product of dialectic among the bio-psychological forces and the socio-historical contexts. In the general context, it is directed towards the development of three stages of work: identifying the perception of changes experienced in the period prior to the study; the perception of the main turning points which mark a significant change in the life course; and the perception of occurrences and socio-historical changes which marked the life of each one^(^ 6 ^)^.

In the present study, the aim was to identify the changes which occurred over the year prior to the study in the life course of older adults from three northeastern capitals, and the values attributed to these changes. 

## Method

This is a multicentric, cross-sectional study, of the inquiry type, undertaken in three cities of the Brazilian Northeast: João Pessoa (in the state of Paraíba); Natal (Rio Grande do Norte) and Teresina (Piauí). The selection of these locations was agreed among the creators of the study and the respective research groups on aging, under an agreement for international cooperation. The pact led to an exchange of information between the research coordinators during an event on CEVI in Geneva. 

CEVI works with various age groups, which are divided in the following way: (1) 20-24 years old: entrance into adult life; 35-39 years old: professional and family life in place; 50-54 years old: advanced professional and family life; 65-69 years old: retirement or third age; 80-84 years old: old age, or fourth age^(^ 7 ^)^.

The research sample in the three capitals was made up of five distinct age groups. These cut-off points correspond to a chronological approximation of positions which are typical in the life course. The noun "approximation" is emphasized, as chronological age is a marker for positions which are subject to spatial-temporal variations.

These criteria may be understood based on the cut-off points used in psychology: the beginning of adult life - understood as being between 18 and 30 years old, a period to be one of transition between professional training and social status; intermediate, understood as being between 30 and 55 years old, a rapid period of life, in which the individuals think that they are preparing to live life; and after 55 years old, late maturity, the tasks in this phase of life being different from the others as they are defensive and preventive^(^ 5 ^,^ 8 ^)^.

Another explanation for the selection of the age groups expresses the generational variations: between 1926 - 1930, 1941 - 1945, 1956 - 1960, 1971 - 1975, and 1990 - 1994. As a consequence, what distinguishes them must not be interpreted only in terms of age position. In the first two stages of the study, the age classes are interpreted principally in terms of position in the life course. In the third stage, they mainly express birth groups (or of generation), that is, belonging to a particular generation^(^ 6 ^-^ 7 ^)^.

Considering the three research locations, it was defined that a minimum of 600 persons would be interviewed. Each city would be responsible for 200 interviews, with 40 in each age group. For this study, two age groups were selected: 65 to 69 years old, and 80 to 84 years old, to total 240 subjects (80 from each city). In the cities of Teresina and João Pessoa, the collection did not present losses. In Natal, the insufficient number of older adults in the age range 80 - 84 years old determined the exclusion of 5 interviews, and the inclusion of one more in the age range 65 - 69 years old.

In order to facilitate data collection, the decision was made to use a non-random sample, stratified by age and sex. The heterogeneity within the groups occurred through the selection of people from different social environments, even without aiming to obtain a representative sample. The older adults were recruited in churches, older adults' groups in primary healthcare centers, third age clubs, retired teachers and residents of economically-favored regions. Only in Natal were older adults recruited who lived in long-term care facilities (LTCP).

The data were collected simultaneously in the three cities between October 2010 and October 2011, with the assistance of a standard CEVI questionnaire, made up of five parts: perception of recent changes (last year) experienced; perception of the principal events which caused changes in the life course; perception of socio-historical occurrences and changes which marked the life of each one; free word association test (TALP) and socio-demographic information. 

For this article, socio-demographic information (sex, marital status, education, self-evaluation of state of health) was used, along with the first part of the instrument with the following question: "During the last year (from January 2010 until now) have there been significant changes in your life?" In the event of affirmative responses, they indicated what these changes were and whether they had represented gains and/or losses. 

The responses to the open questions were transcribed faithfully and in full in the database. The coding and the construction of national databases followed the CEVI standard, in which a pre-coding was prepared in Geneva, with the aim of standardizing the information. The following categories of changes were made: *health *(illness, accident, surgery, hospitalization, psychological health/depression, decline in health, recuperation, other); *family* (births, pregnancies, marriages, divorces, family relationships, other); *death* (deaths, mourning); *activities* (Leisure, sports, participation in special interest groups, other); *profession* (beginning work, promotion/recognition, change of work, leave/unemployment, return to work, retirement, other); *geographical space* (change of city, state or country; entry into a nursing home); *education* (beginning a course, concluding a course, preparing for university entrance, lack of educational success, technical course, returning to study); *economics* (change of economic situation, selling/buying an asset, other).

The data were analyzed using the program "Statistical Package for the Social Sciences" SPSS^(r)^ for Windows^(r)^ version 19.0, and were submitted to statistical treatment through bivariate analysis and the Chi-squared association test (χ^2)^.

The undertaking of the study followed the ethical precepts governed by Resolution 196/96 of the Brazilian National Health Council^(^ 9 ^)^, and was approved by the Ethics Committee of the Lauro Wanderley Teaching Hospital, of the Federal University of Paraíba, under N. 261/09.

## Results

Of the total of 631 subjects interviewed by the study regarding the occurrences over their life (CEVI), 236 individuals were selected, of whom 121 corresponded to the age group between 65 and 69 years old, and 115 corresponded to the age group between 80 and 84 years old, representing 37.4% of the participants. 

In relation to sex, it was ascertained that in the population studied, there was a predominance of females (137/58.1%); among the older adults aged between 65 and 69 years old this proportion was 57.9%, and among those aged between 80 and 84 years old it was 58.5%. In relation to marital status, 47.5% were married/in a stable relationship, and 28.8% were widowed. The condition of widowhood was most significant among the older adults aged between 80 and 84 years old (45.2%). 

Regarding level of education, 43.7% among the older adults aged between 65 and 69 years old, and 58.3% among those aged between 80 and 84 years old, stated that they could read and write. In both groups, illiteracy was below 5.0%. In relation to self-evaluation of health, 57.0% of the older adults aged between 65 and 69 years old stated that their health was positive, as against 33.0% of the older adults aged between 80 and 84 years old. 

In the analysis on the changes which had occurred in the year prior to the study, considering the two age ranges investigated (Table 1), it was observed that 30.0% reported that the principal change in their life course over the last year had been their health, with this category of response being more significant among the older adults aged between 80 and 84 years old (37.7%). 

**Table 1 -:** Changes in life mentioned by the participants for the year prior to the study, by each age range. João Pessoa, Paraíba (PB), Natal, Río Grande do Norte (RN) and Teresina, Piauí (PI), Brazil, 2010-2011

Changes*	Group of older adults						Total		*P^†^*
	65 to 69 years old			80 to 84 years old					
	n	%		n	%		n	%	
Health	31	21.2		63	37.7		94	30.0	<0.001
Family	18	12.3		18	10.8		36	11.5	
Death	12	8.2		18	10.8		30	9.6	
Activities	15	10.3		15	9.0		30	9.6	
Profession	23	15.8		2	1.2		25	8.0	
Geographical space	9	6.2		11	6.6		20	6.4	
Travel	5	3.4		13	7.8		18	5.8	
Self	9	6.2		8	4.8		17	5.4	
Education	9	6.2		4	2.4		13	4.2	
Economics	5	3.4		4	2.4		9	2.9	
Friendships	1	0.7		3	1.8		4	1.3	
Various	9	6.2		8	4.8		17	5.4	

* The categories correspondent to this variable allowed multiple response options

† Chi-square = 35.391

The occurrence of changes in the family environment was mentioned by 12.3% of the older adults aged between 65 and 69 years old, and by 10.8% for those aged between 80 and 84 years old. After that, the following were mentioned: death (9.6% of the total) and changes in routine activities (9.6% of the total). 

Emphasis is placed on specific characteristics between the age groups, evidenced in the "profession" category, as being a change for 15.8% of the older adults aged between 65 and 69 years old, and in the "death" category among the older adults aged between 80 and 84 years old (10.8%). Statistically, there was a significant difference (p<0.001) between the two groups when they were analyzed in relation to the changes mentioned. 

Regarding the valuing of the changes which had occurred in the year prior to the study (Table 2), it is observed in the group aged between 65 and 69 years old (64.7%) that the changes had brought gains to the older adults. For 49.4% of the older adults who were older, the changes were related to losses. In the comparison between the valuing of the changes and the age groups, it was ascertained that there was a statistically significant difference (p=0.003) between the groups compared. 

**Table 2 -:** Distribution of the valuing of changes between the age ranges. João Pessoa (PB), Natal (RN) and Teresina (PI), Brazil, 2010-2011

Valuing of the Changes	Group of older adults						Total		P *
	65 to 69 years old			80 to 84 years old					
	n	%		n	%		n	%	
Gain	86	64.7		71	43.8		157	53.2	0.003
Loss	43	32.3		80	49.4		123	41.7	
Both	4	3.0		9	5.6		13	4.4	
Neither one nor the other	-	-		2	1.2		2	0.7	
Total	133	100.0		162	100.0		295^†^	100.0	

* Chi-square = 13.768 †A total of 18 missing was identified among the valuations, there were older adults who did not answer this question, hence the difference between these values and Table 2

## Discussion

One of the study's proposals was to maintain the stratification between the groups; however, this is not possible, as among the older adults, there was a predominance of the female sex. This predominance is a striking characteristic of aging in Brazil^(^ 10 ^)^.

The level of education differed from other studies undertaken in the Northeast region, which indicated that on average 60% of the older adults had not attended school^(^ 11 ^-^ 12 ^)^. These data were confirmed by studies undertaken by the Brazilian Institute of Geography and Statistics (IBGE) in the Brazilian Northeast, in which high rates of illiteracy were observed among older adults^(^ 13 ^)^. The possible explanation for this fact is due to opportunities for studying which the younger generations of older adults had, differing from the older cohort of older adults. At the present time there is much encouragement for the promotion of literacy in the so-called third age, and the elections were also a mark in this campaign for reducing illiteracy, as - in order to vote - the older adults learned to read and write their names on the ballot papers.

In this study, the self-evaluation of health presented a higher prevalence in relation to being positive among the older adults aged between 65 and 69 years old, in comparison with those aged between 80 and 84 years old and also in comparison with studies which focus on this evaluation. In Bahia, where the mean age was 75 years old, and there was no differentiation between the sexes in the self-evaluation, the majority (51%) mentioned their state of health as good or very good^(^ 14 ^)^. In Spain, on the other hand, the female older adults had a more negative perception of their health in comparison with the men, indicating that with advancing age, the self-evaluation of health changes, being less perceptible over the years, than the actual health condition^(^ 15 ^)^.

The difference in the perception of the state of health is owed to the fact that the sample works with two distinct groups of older adults in different contexts. In Spain^(^ 15 ^)^ the researchers investigated female older adults in long-term care facilities, and in Bahia they investigated older adults attended in health services^(^ 14 ^)^. When the older adults evaluated their state of health, many associated their physical limitations and chronic degenerative diseases as the reference point for good or bad health conditions. Generally speaking, the incapacitating illnesses are responsible for the negative view which is held of aging. 

When the CEVI study was undertaken in Switzerland, the older adults aged between 65 and 69 years old mentioned having a good condition of health, and even though the country is very different from Brazil, this was similar to the findings of the study held in the Brazilian context; the Swiss older adults aged between 80 and 84 years old, however, have a different perception, reporting their health as satisfactory, a view which - in Brazil - is more negative in this age range^(^ 6 ^)^.

Comparing a country said to be in development with another - which is considered to be developed - raises various questions regarding the self-perception of health of the older adult, which is influenced by multiple factors, among which one can identify: age, sex, family support, conjugal status, level of education, socio-economic condition, chronic health conditions, lifestyle and functional capacity^(^ 16 ^)^.

The present study identified various types of changes in the older adults' lives, and among those mentioned most in the year prior to the study, questions were involved related to health conditions, a similar result to that found in Switzerland^(^ 6 ^)^. It is frequent for the older adults to mention, as changes, events involving health, interpersonal and financial relationships, and those related to work, these events being more prone to affecting emotions or causing the older adults to develop strategies for coping with the illness^(^ 17 ^)^.

It is important to emphasize that the events are not related exclusively to the older adults, but to their close family members, such as, for example: wife/husband, grandson/daughter, and son or daughter. 

Studies argue that advanced age presents a higher probability of physical and health problems, of pain, of musculoskeletal problems, among others, which lead to indisposition, low energy and fatigue among older adults, leading to compromise of activities of daily living^(^ 18 ^)^.

The gradual decline in health is part of the aging process, and the older adults believe that, in aging, their health conditions tend to worsen, and that the older one is, the more predisposed one is to present illness than are younger people. In this process, the family exercises an important social role, as it frequently becomes essential for promoting care or assistance in the older adult undertaking activities of daily living^(^ 11 ^)^.

It is not only when they become ill that the older adults remember the family; events such as births, weddings and other family celebrations were reported. In the family, the individual perception regarding certain changes, such as, for example, lifestyle, becoming ill, or quality of life can influence the family dynamic. The older adult's progressive weakness, the dependence for care, creates impacts on the intrafamily relationships, which have to cope with the new^(^ 18 ^)^, and create adaptation mechanisms.

Social support is a coping resource obtained from interpersonal relationships, whose effects include the maintenance of health and the reduction of vulnerability to physical and mental illnesses in older adults. The interpersonal relationships facilitate coping with stressful situations in the life of older adults who live in the community or who are in long-term care facilities^(^ 2 ^,^ 19 ^)^. Generally speaking, it is the adults who live alone who tend to report less social support and more solitude, and who consequently experience an accelerated cognitive decline^(^ 20 ^)^. 

In Argentina, in one study on life trajectories, also based in CEVI, situations such as death and mourning were those which most affected family life, a fact generally expected in this stage of life^(^ 21 ^)^. In this same study, the older adults prioritarily mentioned occupation as being the most important change. This was significantly different from the reality of the Brazilian and the Swiss results. 

Death does not need to be linked only to the family to be a significant event in the life of older adults. One study evidenced that the loss of close friends performs a special role in the life of female older adults, as friendship is important in maintaining psychological well-being and mental health^(^ 20 ^)^. Both death and friendship are events mentioned as significant changes for the groups of older adults in the study. 

The majority of the studies would agree that the process of aging is characterized by a change in the balance between gains and losses. As people age, this ratio becomes less favorable, as the losses can be constant in various areas of life of the individual, including physical, mental, and health^(^ 22 ^)^.

Contradicting another study, the results showed that the older adults of the two groups presented gains, as for the older adults aged between 65 and 69 years old, there were not so many negative changes, while among those aged between 80 and 84 years old, losses were slightly above the gains, although this does not mean that they only had negative occurrences in their lives. The results found in this study are similar to those found in Switzerland, where there is greater life expectancy and better socio-economic conditions^(^ 6 ^)^.

Remembering and valuing events which occurred in life is one way of encouraging the older adult to use his or her memory. When the exercising of memory is encouraged, the individual has the opportunity to relive events which had greater meaning in his life, which memories may be full of with pleasure and happiness^(^ 23 ^)^. It is at this time that the individual allows their life to be told and their problems to be exposed. 

It is important for health professionals and nursing professionals to recognize that the happenings or changes over life express the process of life, of aging, and of reconstruction. Each point of life differs in accordance with the dimension which affected it, whether positively or negatively. Change, in particular when there are many losses, can be more dramatic and can cause to be submerged how fundamental the family is as a closer social unit of support in this phase of transition. 

It is the life experiences, and how they affect the routine, that will say whether they are significant, or not, to the extent of being mentioned later, and their valuing. In the universe of aging, there are various changes which each older adult has to face, whether in their social, biological, financial or matrimonial life; at the end of the day, they are new situations, which were grasped by this study and which are of extreme relevance, as it is based on this that one can make further questions and trigger reflections which were not addressed by this study. 

This study's findings contribute to helping the professionals in the care of older adults, whether during an intervention or other type of attendance, evidencing the importance of encouraging the older adult's memory, mental and physical exercises, care with health, and principally the social relationships.

## Conclusion

The results showed that the changes found between the two groups of older adults from three Northeastern Brazilian cities converged in both the age ranges, in relation to the changes in the health conditions determined by aging, with greater significance among those older adults aged from 80 to 84 years old. Although it was not an objective of this study, it is worth emphasizing that such results diverge in some aspects from the other two countries in which the CEVI standard questionnaire was applied. In Switzerland, the In Switzerland, the older cohort mentioned greater satisfaction relating to their health conditions. This observation allows one to indicate that differentiated contexts and standards of social organization and of the healthcare network walk side-by-side with the (in)ability to age healthily. 

As the first study of this nature in Brazil, it is emphasized that health professionals' understanding in relation to the valuing of changes caused in the life course, and of how these affect the routine or the health-illness process of these individuals can change and determine strategies for coping with certain situations during human aging. 

It is recommended, therefore, that further studies should be undertaken in this area so as to better elucidate the questions raised here, principally in relation to the changes in these subjects' lives, and to draw attention to the perception which the older adults have in relation to the health condition, which requires health policies which are adapted to the specific characteristics. 

The limitations of the sample, and the selection by convenience, did not compromise the results, however, they must be taken into account in future studies, so as to allow generalization of the information. The variables studied did not demonstrate significant differences between the three northeastern capital cities. The scarcity of studies of this nature hindered comparisons in greater depth. 
